# Analyst coverage and manufacturing enterprise green transition: An empirical study based on Chinese enterprises

**DOI:** 10.1371/journal.pone.0297851

**Published:** 2024-01-30

**Authors:** Jianfei Leng, Jianqin Hu

**Affiliations:** Business School, Hohai University, Nanjing, China; Cavendish University / Kyambogo University, UGANDA

## Abstract

This study used the entropy weight method to develop an index of green transition and empirically examined the influence of analyst coverage on green transitions by manufacturing enterprises in China. We examined A-share listed manufacturing firms from 2010–2020, using patent data, media reports from Chinese Research Data Services, and other data from the Cathay Capital Database. After excluding cases with missing data, our final sample comprised 16,576 observations. The following conclusions were drawn. First, analyst coverage significantly contributed to green transition. Second, the analysis of the impact mechanism showed that improving information transparency, weakening principal-agent conflict, and increasing environmental legitimacy pressure are the paths through which analyst coverage affects manufacturing’s corporate green transition. Third, the effect of analyst coverage was stronger for large-scale and state-owned manufacturing companies.

## 1. Introduction

Since the reform and opening up, the Chinese economic development model of “emphasizing efficiency and neglecting environmental protection” has created a serious burden on the environment. The resource consumption and environmental constraints caused by the traditional industrial growth model have limited economic growth and hindered the high-quality development of manufacturing. China urgently needs to change its development mode to realize the sustainable development of the manufacturing industry. The green transition is the inevitable choice for sustainable development. “Made in China 2025” explicitly proposes encouraging the green transition and upgrading manufacturing through research and development (R&D) on leading-edge energy-saving and environment-friendly technologies, processes, and equipment. Green transition refers to a process whereby enterprises prioritize green development and adopt green technology to improve the environment and facilitate sustainability [1–3]. However, faced with the realistic constraints of environmental protection and limited resources, many manufacturing enterprises lack the motivation to undertake a green transition. This trend can be explained by two reasons. First, the green manufacturing technology level of most manufacturing enterprises in China is low, and the proportion of traditional resource inputs is high. Second, green transition requires extensive upfront costs, and the government’s environmental regulations will increase the production and operation costs of enterprises. Some manufacturers, particularly private enterprises, cannot take the initiative in green transition owing to cost pressures. Therefore, it is imperative to discuss the factors that promote the green transition of Chinese manufacturing.

Many scholars believe that governments play an indispensable role in promoting the green transitions of enterprises [4]. Some point out that governments can encourage green transition by introducing environmental subsidy policies [5, 6], strengthening environmental regulation [7, 8], and promoting cleaner production technologies [9]. Additionally, some local governments have established carbon trading markets to reduce the impacts of global climate change [10]. However, some difficulties still hinder the government’s work to promote corporate green transition and, more broadly, China’s environmental governance. First, government subsidies do not always play a positive role in promoting enterprises’ green transitions. Moreover, differences in the government’s policy support for different industries drive the “policy arbitrage” behavior of enterprises and do not substantively promote their green transitions [11]. Second, because some polluting enterprises serve as tax sources for local governments, local governments may collude and engage in rent-seeking behavior with polluting enterprises for economic benefits [12]. Additionally, according to neoclassical economics, environmental regulation increases firms’ compliance costs, squeezes the resources that firms use for green innovation, and increases their financial burden, limiting their productivity and market competitiveness [13, 14]. Therefore, promoting the green transitions of enterprises is a wide-ranging and long-lasting systematic project that requires large investments—relying solely on government subsidies and regulation is unsustainable. Multi-party co-management, in particular, plays a necessary supervisory role in the market.

Financial analysts, as an important stakeholder group, are experts in evaluating and advising on specific firms [15, 16]. They are widely recognized as key information intermediaries in the capital market and play a significant role in collecting, processing, and disseminating relevant market and firm information [17]. With the rapid emergence of financial markets during China’s social transition, the analyst community has been expanding, the level of standardization has been increasing, and academic discussions on analysts have been growing. Some studies support analysts conducting research on their covered firms and disseminating their findings to the public, arguing that this work lowers the information asymmetry between firms and investors and monitors firms’ performance. Lin et al. [18] find that information spreads more slowly across financial markets for weakly covered firms. Mehran [19] and Mola [20] find that analyst coverage significantly increases the probability of going public because companies with higher coverage are recognized by more investors. Kim [21] finds that being followed by an analyst can reduce a firm’s stock overevaluation regarding financial markets and improve investors’ confidence, thus reducing the probability of extreme events and future stock price crashes. Moreover, analysts provide monitoring and reduce conflicts of interest and agency problems. Dyck et al. [22] report that analysts reveal 16.9% of corporate fraud cases, which is higher than the 11.3% for auditors. This suggests that analysts play a more important role than the SEC and auditors in overseeing firms’ financial reporting. Chen et al. [23] and Yu [24] show that when only a small number of analysts focus on a firm, the firm’s management is more likely to invest in value-destroying projects and actively engage in surplus management strategies. In contrast, there is a view that analysts have less access to company-specific information and are also subject to complex external interests that can have a limited or even negative effect on the business activities of listed companies. Some researchers have found that financial analysts are company outsiders whose forecasts rely more on market- and industry-wide information, resulting in analyst-focused companies exhibiting higher stock return synchronization [25]. Some analysts are subject to various conflicts of interest and have a severe selective bias in the information they convey to the market. They tend to issue optimistic surplus forecasts and stock ratings, a behavior that will lead management to act more aggressively and even make larger discretionary accruals to meet the forecasts. When analysts’ optimistic bias makes it difficult to disclose negative news about the company to outside investors in a timely way, this accumulation and eventual release of negative information may lead to a significant drop in the stock price and the risk of a crash [16, 26]. Therefore, we further explore the impact between analyst coverage and green transition, which has important practical implications for corporate strategy.

Against a background where green transition and ecological protection are increasingly becoming the world’s main themes, can analyst coverage and performance forecasting impact green transition? Based on the findings of the literature, we argue that analysts theoretically have two diametrically opposed effects on corporate green transition. Based on the role of information disclosure and monitoring, analysts can improve the information content of stock prices, improve the efficiency of capital market operations, and mitigate agency problems, which can, in turn, positively impact green transitions [27–29]. Specifically, green transition is typically a positive green activity that will be an instrumental part of analysts’ assessment of firm value and forecast performance. Simultaneously, managers typically have an incentive to cater to analysts’ preferences. Therefore, firms that are covered by more analysts have a greater incentive to engage in green transition activities. In this way, analysts drive firms to green transition by communicating to outsiders that firms are less likely to be penalized for polluting and more likely to capture the booming green market. By contrast, according to the market pressure hypothesis, the earnings forecasts issued by analysts may place short-term performance pressures on managers and aggravate the principal-agent conflict of listed companies, which may, in turn, adversely affect green transitions [30, 31]. At this stage, green activities not only signal pro-social behavior, but are also an important source of information in external evaluations of organizations. There is an urgent need to open the black box connecting analysts and corporate green transition by identifying potential mechanisms for the conflicting speculations that analysts may have on corporate green transition. However, little literature has focused on the impact of analyst coverage on corporate green transition. This study contributes to understanding the effect of financial analysts on green transition by testing the above two effects in a unified framework.

Another empirical issue relates to the measurement of green transition. Largely, studies have used green total factor productivity or green innovation as a green transition benchmark. Liang et al. [32] focus on the green transition of firms from an innovation perspective characterized by green patents; Wang et al. [33] measure the green transition of firms based on the level and dynamic evolution of green total factor productivity. However, according to the enterprise, green transition is related to the whole industrial chain, the subversion and upgrading of the institutional framework, production process, product design, and other links. It needs to deal with the relationship between the enterprise and nature, within the enterprise, and between the enterprise and society. Therefore, to comprehensively measure firms’ green transition, we construct an index system including green innovation, green production, green governance performance, and social responsibility, and adopted the entropy weight method to measure the green transition of enterprises.

This study makes the following contributions. First, we put analyst coverage into a productive dialogue with scholarship on green transition. This study demonstrates the impact of analyst coverage on firms’ green transitions through theoretical and empirical tests. It addresses the ongoing debate about whether the information effect of analysts outweighs the effect of the pressure they exert on managers. Compared with developed countries, China has a weak investor protection environment. Therefore, the relationship between analyst coverage and corporate green transformation is more likely to depend on the “monitoring effect” of analysts than the “pressure effect.” Additionally, due to the short history of China’s stock market, Chinese stock investors are inexperienced and usually rely on professional information analysts to process information. In this regard, analysts play a profound role in reducing information asymmetry and inequality between managers and outsiders. While previous studies have found evidence that analyst coverage has a significant impact on a variety of corporate decisions, such as financing choices [34], investment choices [35], innovation strategies [17, 31], and accounting and tax policies [24, 36], to the best of our knowledge, no studies have yet analyzed the relationship between corporate green transition and analyst coverage. Nevertheless, financial analysts, as special external institutions, may enable corporate green transition. In response to this gap in the literature, this study builds a theoretical foundation for the impact of analyst coverage on green transition and enriches the real economic consequences of financial analysts in the area of corporate environmentally friendly behavior. Second, we present information and monitoring channels to explore potential explanations for how analyst reporting can contribute to firms’ green transitions. Specifically, as an information intermediary, analyst coverage promotes green transitions by increasing information transparency or reducing information asymmetry. Simultaneously, analytical reporting, as an information intermediary, fulfills an important monitoring function, increasing the pressure on corporate environmental legitimacy by reducing principal-agent conflicts between shareholders and managers and increasing the pressure on corporate environmental legitimacy. Third, we fully explore the heterogeneous impact of analyst coverage on the green transitions of firms of different sizes and equity structures. Manufacturing firms with different firm sizes and equity structures have large differences in resource endowments and may be covered by analysts to different degrees. This work strongly supports the long-term development of manufacturing firms in a green environment.

## 2. Theoretical analysis

The Chinese Communist Party and the state attach great importance to environmental protection. At present, environmental protection lags far behind economic and social development, posing a serious threat to sustainable development. Promoting green transitions in manufacturing is necessary to achieve sustainable economic development in China. China has a unique situation regarding the promotion of green transition. It mainly relies on government regulation. However, governments are limited by administrative resources and are often at an information disadvantage compared to enterprises. Moreover, because local government officials must compete for promotions in China, they prioritize economic development over environmental governance, as economic indicators are more visible and accessible than environmental indicators [37]. The inherent limitations of government supervision allow space for rent-seeking through enterprises’ emission behavior. Government power is insufficient for promoting green transitions; it must be supplemented by market supervision, legal administration, science, and technology. It is crucial to study whether analyst coverage promotes the green transition of enterprises. Through the information transmission effect [38], analyst coverage can enhance investors’ willingness to support green transitions by enterprises. Prior studies have shown that a larger number of analysts following a listed company means that the company has more standardized information disclosure, greater transparency, and lower potential risks, which can attract more investors [39]. Considering that investors’ investment is oriented by financial returns, firms’ green transition activities have positive externalities. In addition, the core of corporate green transition lies in green technology innovation, which has a longer investment cycle and lower success rate compared with general innovation activities. Moreover, green technology innovation allows enterprises to build differentiated competitive advantages. Thus, enterprises typically disclose only a small amount of information about such innovations, intensifying information asymmetries in the market. This leads to the undervaluation of green technology innovation and less willingness among investors to support enterprises’ green transitions. As a key market intermediary, analysts have professional knowledge and sufficient time to obtain information and transmit it to investors in the form of earnings reports. This alleviates the information asymmetry between investors and listed companies, enables the capital market to value green innovation activities more accurately, and enhances investors’ willingness to invest in corporate green transitions.

Through the external monitoring effect, analyst coverage can influence corporate green transitions in two ways. First, coverage reduces the principal-agent conflict. Green transitions are often accompanied by large investments in R&D, which give management more room for rent-seeking and intensify the agency conflict between shareholders and management [40]. Healy and Palepu [41] showed that analysts can conduct direct supervision of managers through research, interviews, and other means. Analysts are also able to use their professional advantages to interpret corporate financial information and discover hidden problems in corporate financial reports, which helps curb management’s opportunistic behavior and regulate corporate operations. Against this backdrop, analysts can effectively supervise managers’ opportunistic behavior and surplus management in the green transition process, weaken corporate principal-agent conflicts, and improve resource allocation efficiency, which promotes corporate green transitions.

Second, analyst coverage increases the pressure on environmental legitimacy. Environmental legitimacy plays an important strategic role in corporate development, helping companies obtain external resources, such as government green subsidies, soft loans, and corporate reputation, through their “environmental premium” [42]. The business model of research ensures a strong connection between analysts and listed companies; analysts can grasp relevant information about listed companies, making it easier to discover corporate environmental violations. Once analysts disclose that a listed company has environmental violations, the firm suffers reputational damage and may face administrative or criminal penalties or even a stock price collapse. As a result of this “deterrent” effect, companies are motivated to embrace environmental legitimacy and adopt proactive environmental management practices and green innovation activities. Analyst coverage also exposes enterprises to the supervision of more market participants; in this respect, studies show that having more analysts track enterprises strengthens supervision [17]. To demonstrate the legitimacy of their behavior and to meet the environmental demands of stakeholders, enterprises must make green changes in their innovation, production level, governance, and social responsibility.

Therefore, based on the information transmission effect and external monitoring effect, we propose the following hypothesis:

H1a: *Analyst coverage is positively associated with the green transition of enterprises*.

The market pressure hypothesis suggests that analysts have a general tendency to be optimistic, failing to perform their true market function and instead placing undue short-term performance pressure on managers [43]. Analysts screen and organize financial and non-financial information released by companies and then produce company surplus forecast reports and stock ratings. If the actual performance of the company does not meet the short-term expectations, the market signals that the company is not operating well, which adversely affects it. As such, management uses surplus management tools, such as dropping items with positive net present value and cutting R&D expenditure, to meet analysts’ surplus expectations [44]. Scholars have confirmed the role of pressure; as more analysts follow listed companies, the pressure on management increases, leading to a decrease in their tolerance for short-term failures, which reduces corporate investment and innovation activities [30, 45]; corporate green transitions rely mainly on green technological innovation. For these reasons, we believe that biased surplus forecasts issued by analysts can put short-term performance pressure on management, forcing them to implement surplus management proactively. This may reduce corporate transparency and increase information asymmetries. Driven by performance pressure, firms that are tracked more by analysts may reduce corporate venture capital and green innovation activities, which hinders their green transition.

Therefore, based on the market pressure hypothesis, we propose the following hypothesis:

H1b: *Analyst coverage is negatively associated with the green transition of enterprises*.

**Fig 1:**
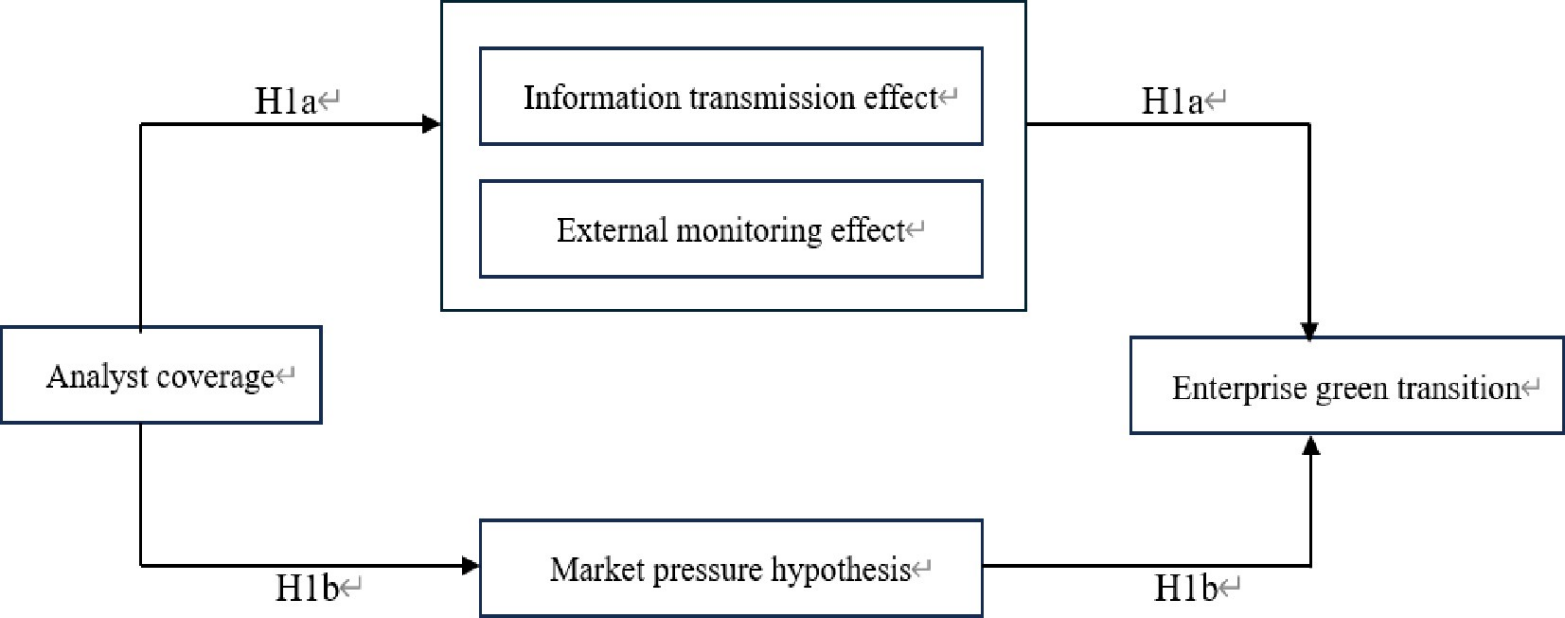
Theoretical framework diagram.

Fig 1 shows a summary of this paper’s theoretical model.

## 3. Research design

### 3.1. Sample selection and data sources

Our sample consisted of A-share listed manufacturing companies for the years 2010–2020. For these firms, we collected patent data and media reports from Chinese Research Data Services (CNRDS), and the remaining data are from the Cathay Capital Database (CSMAR). After excluding cases with missing data, our final sample consisted of 16,576 observations. To reduce the influence of outliers, the relevant continuous variables were Winsorized at the upper and lower 1% levels.

### 3.2. Definitions and measurements of variables

#### 3.2.1. Analyst coverage and enterprise green transition (EGT)

Following Martens and Sextroh [46], we used the natural logarithm of the number of analysts tracking the target firm plus one as a proxy variable for analyst coverage.

#### 3.2.2. Enterprise green transition

Enterprise green transition should consider production efficiency and environmental protection [47]. However, it is difficult for a single index to measure the two factors concurrently. Therefore, we followed Lu et al. [48] and Sun and Zhang [49] and constructed an evaluation system for enterprise green transition focusing on four items: green innovation, green production, green governance performance, and social responsibility. Through the entropy weight method [50], on the basis of analyzing the degree of correlation between indicators and the amount of information provided by indicators, the evaluation indicators were objectively assigned to avoid the bias caused by subjective factors. Meanwhile, we used corporate green innovation as a proxy indicator for green transition for robustness testing. Below, we define the indicators and their measures.

1. *Green innovation*. The main driving force behind the green transition of enterprises is green innovation. It consists of improving technical capacity through independent R&D or outsourcing green technology to avoid risks and realize sustainable development. It includes innovation input and output, which we measured using the proportion of R&D funds invested by enterprises and the ratio of green patents granted to the total number of patents granted, respectively.
2. *Green production*. Green production means the transition of enterprises from traditional production to methods with lower environmental impact and higher labor and production efficiency. Production efficiency was measured by total factor productivity, while labor efficiency was measured by the ratio of total business revenue to the number of employees.
3. *Green governance performance*. Existing studies define this variable as green actions taken by enterprises based on environmental governance, and most studies use environmental information disclosure to measure performance [51]. Therefore, we used the disclosure of corporate pollution management, environmental management, environmental regulation, and cleaner production facilities in our evaluation of green transitions. Specifically, we included wastewater, waste gas, and solid waste treatment; environmental management systems, such as emergency mechanisms and the “three simultaneous” system (this is the earliest environmental management system in China; notably, it requires all environmental protection facilities configured in a construction project be designed, constructed, and put into operation at the same time as the main project); details of clean production facilities; environmental information disclosure; environmental violations; and environmental management system certification. If the relevant information was disclosed, the variable was coded 1; otherwise, it was coded 0.
4. *Social responsibility*. Enterprise green transition involves not only the relationship between the firm and nature but also the relationship between the firm and society [52]. Therefore, we incorporated corporate social responsibility performance into our evaluation system using the Hutchinson Social Responsibility (CSR total score).

The specific steps are shown as follows:

First, the indicators are normalized.

For positive indicators *x*_*ij*_, set $x_{ij}^{\prime }=\frac{x_{ij}-minx_{ij}}{maxx_{ij}-minx_{ij}}$

For negative indicators x_ij_, set $x_{ij}^{\prime }=\frac{maxx_{ij}-x_{ij}}{maxx_{ij}-minx_{ij}}$

ⅰ = 1,2, …n; j = 1,2, …m. *i* denotes the number of samples and *j* represents the number of indicators.

Second, we calculate the proportion of the *j*_*th*_ indicator under the *i* system *p*_*ij*_: $p_{ij}=\frac{x_{ij}^{\prime }}{\sum_{i=1}^{m}x_{ij}^{\prime }}$

Third, we calculate the proportion of the *j*_*th*_ indicator *e*_*ij*_: $e_{ij}=-k\sum_{i=1}^{m}p_{ij}lnp_{ij}$. Where the adjustment factor *k* = 1/*lnm* >0, if *p*_*ij*_ = 0, then definite $\underset{p_{\mathrm{ij}}\to 0}{\lim}p_{ij}lnp_{ij}=0$

Fourth, we calculate the weight of the *j*_*th*_ indicator *w*_*j*_: $w_{j}=\frac{1-e_{j}}{\sum_{j=1}^{n}\left(1-e_{j}\right)}$

Finally, we calculate the composite index of all lever indicators *z*: $z=\sum_{j=1}^{n}w_{j}x_{ij}^{\prime }$

#### 3.2.3. Control variables

Referring to previous studies, we controlled for a rich set of firm and industry characteristics that may possibly affect enterprise green transition, including profitability, ownership form, enterprise age, rights of shareholders, board size, fixed asset ratio, enterprise growth capability, cash flow level, and book-to-market ratio [53]. Table 1 reports the definitions of the variables.

**Table 1:** Main variables and definitions.

Variable name	Variable definition
Enterprises green transition	Enterprise Green Transition Index
Analyst coverage	Ln (total number of analysts tracking an enterprise + 1)
Analyst	The number of analysts per year of a firm
Profitability	Net profit/total assets
Ownership form	State-owned enterprise or not, if yes, take 1; otherwise, take 0
Age	Ln (The establishment period of the enterprise)
Rights of shareholders	Shareholding ratio of top ten shareholders
Board size	Number of board members
Fixed assets ratio	Fixed assets/total assets
Growth capability	(Revenue of the current period-revenue of the previous period)/revenue of the previous period
Cash flow level	Net cash flow from operating activities/total assets
Book-to-market ratio	Net assets/market value of the company

### 3.3. Model setting

To investigate the impact of analyst coverage on enterprise green transition, we specified the following model using OLS.


$$EGT_{i,t}=\beta_{0}+\beta_{1}Analyst_{i,t}+\rho_{k}X_{i,t}+\mu_{i}+\gamma_{t}+\varepsilon_{i,t}$$ (1)

The subscripts *i* and *t* represent enterprise and year, respectively. *EGT*_*i*,*t*_ is the level of enterprise green transition. *Analyst*_*i*,*t*_ stands for analyst coverage. *Β*_*0*_ is the intercept term; *β*_*1*_ indicates the impact of analyst coverage on enterprise green transition; *X*_*i*,*t*_ is a vector of control variables; *μ*_*i*_ represents the year fixed effect; *γ*_*t*_ represents the industry fixed effect; and *ε*_*i*,*t*_ is the error term. Standard errors are robust to heteroskedasticity and clustered at the firm level.

## 4. Empirical results

### 4.1. Descriptive statistics

Table 2 shows that the maximum, minimum, and mean values of enterprise green transition were 0.611, 0.069, and 0.224, respectively. This indicates that the level of green transition among enterprises in China was low and uneven. The mean value of analyst coverage was 7.523, and the median value was 3, which means that most of the manufacturing companies received the attention of at least three analysts. The maximum value of analyst coverage was 75, and the minimum value was 0, indicating that the number of analysts varied widely among companies. The values of the control variables—profitability, ownership form, enterprise age, rights of shareholders, board size, fixed asset ratio, enterprise growth capacity, cash flow level, and book-to-market ratio differed significantly between firms.

**Table 2:** Descriptive statistics.

Variable	N	Mean	P50	Sd	Min	Max
Enterprise green transition	16576	0.224	0.205	0.086	0.0690	0.611
Analyst coverage	16576	1.487	1.386	1.186	0.000	4.331
Analyst	16576	7.523	3.000	9.982	0.000	75.000
Profitability	16576	0.042	0.042	0.061	-0.242	0.199
Ownership form	16576	0.280	0.000	0.449	0.000	1.000
Age	16576	1.858	1.946	0.925	0.000	3.367
Rights of shareholders	16576	59.263	60.345	14.531	24.65	88.790
Board size	16576	8.476	9.000	1.580	0.000	18.000
Fixed assets ratio	16576	0.226	0.201	0.135	0.000	0.872
Growth capability	16576	0.183	0.094	0.588	-0.886	3.089
Cash flow level	16576	0.049	0.048	0.072	-1.938	0.664
Book-to-market ratio	16576	0.414	0.353	0.262	0.062	1.395

### 4.2. Benchmark regression

We estimated a regression of Eq 1, and Table 3 presents the results. In column 1, the model in which control variables are not included, the coefficient for analyst coverage was 0.016 (p<0.01), which supports H1a. After including the control variables (column 2), the regression coefficient for analyst coverage was 0.0125 (p<0.01). This indicates that firms with high analyst coverage will have higher levels of green transition. The economic magnitude of these findings is also significant. Specifically, with a one standard deviation (0.0011) increase in analyst coverage, the level of corporate green transition increases by 0.06% (0.0125 × 0.0011 ÷ 0.224). This finding supports H1a.

**Table 3:** Benchmark regression of corporate green transition on analyst coverage.

	(1)	(2)
	Enterprises green transition	Enterprises green transition
Analyst coverage	0.0160***	0.0125***
	(0.0011)	(0.0011)
Profitability		0.1451***
		(0.0169)
Ownership form		0.0137***
		(0.0038)
Age		0.0140***
		(0.0018)
Rights of shareholders		0.0005***
		(0.0001)
Board size		0.0052***
		(0.0009)
Fixed assets ratio		0.0635***
		(0.0110)
Growth capability		-0.0041***
		(0.0011)
Cash flow level		0.0806***
		(0.0137)
Book-to-market ratio		0.0438***
		(0.0053)
Constant	0.2005***	0.0627***
	(0.0019)	(0.011)
Industry	Yes	Yes
Year	Yes	Yes
N	16576	16576
R^2^	0.2025	0.2818
Adjusted R^2^	0.1993	0.2783

Note

*p < 0.1

**p < 0.05

***p < 0.01

Regarding the control variables, profitability significantly increased the enterprise green transition, indicating that green transitions are resource-intensive for firms and dependent on favorable firm performance. Form of ownership had a significant positive relationship with enterprise green transition, which may be due to state-owned enterprises being more closely related to the government and thus required to assume more social and environmental protection responsibilities. Shareholder rights and board size positively influenced enterprise green transition, which may be because both shareholders and the board of directors play supervisory roles, inhibiting short-sighted behaviors that may adversely affect green transitions. Meanwhile, the regression coefficients of firm age, cash flow level, and fixed asset ratio were all significantly positive. These findings indicate that firms that had been in operation longer had higher cash flow and fixed asset ratios and were more likely to engage in green transitions. Further, growth capability had a significant negative impact on green transitions. Still, the book-to-market ratio positively influenced green transitions, suggesting that companies with strong liquidity and high growth potential were more inclined to undergo green transitions.

### 4.3. Endogeneity test

#### 4.3.1. Instrumental variable approach

There may be an inverse causal relationship between analyst coverage and enterprise green transition. Our results in Table 3 suggest that analyst coverage promotes corporate green transition through its information transmission mechanism and external monitoring mechanism; conversely, information related to green innovation, green production, green governance, and social responsibility disclosed in the process of corporate green transition will also attract more analyst attention to the company. To address this potential endogeneity issue, we constructed an instrument to capture the variation in analyst coverage exogenous to firms’ green transition and used 2SLS for regression. As Yu [24] argued, the size of a brokerage firm, which means the numbers of analysts employed by brokerages usually depend on their performance or profitability, is unlikely to cover firms’ green transition. Therefore, the change of analyst coverage driven by the difference of brokerage institutions could be a valid instrumental variable. To generate the expected coverage of as many firm-year observations as possible, we used 2015, the middle year of the sample period from 2010 to 2020, as the base year. The expected analyst coverage instrument variables were constructed as follows:

$${Expcoverage}_{i,t}=\sum_{j=1}^{n}(\frac{{Analyst}_{t,j}}{{Analyst}_{0,j}})\times {coverage}_{i,0,j}$$ (2)

where *Analyst*_*t*,*j*_ and *Analyst*_*j*,*0*_ are the number of analysts employed by broker *j* in year *t* and in year 0, respectively. *Coverage*_*i*,*0*,*j*_ is the number of analysts from broker *j* following firm *i* in year 0. *Expcoverage*_*i*,*t*_ is the expected coverage of firm *i* in year *t*.

As shown in columns (1)–(2) of Table 4, the regression coefficient for analyst coverage instrumented by expected coverage is 0.0207 (p<0.01), which is consistent with the previous finding.

**Table 4:** Endogeneity test.

	(1)	(2)	(3)
	First stage	Second stage	Enterprises green transition
	Analyst Coverage	Enterprises green transition	
Expected coverage	0.4395***		
	(0.0075)		
Analyst coverage		0.0207**	0.0113***
		(0.0031)	(0.00134)
Constant			0.0829***
			(0.0131)
Control variables	Yes	Yes	Yes
Industry	Yes	Yes	Yes
Year	Yes	Yes	Yes
N	6560	6560	5721
Cragg-Donald Wald F statistic	6324.50		
R^2^		0.1636	0.2720
Adjusted R^2^			0.2636

Note

*p < 0.1

**p < 0.05

***p < 0.01

#### 4.3.2. Placebo test

**Fig 2:**
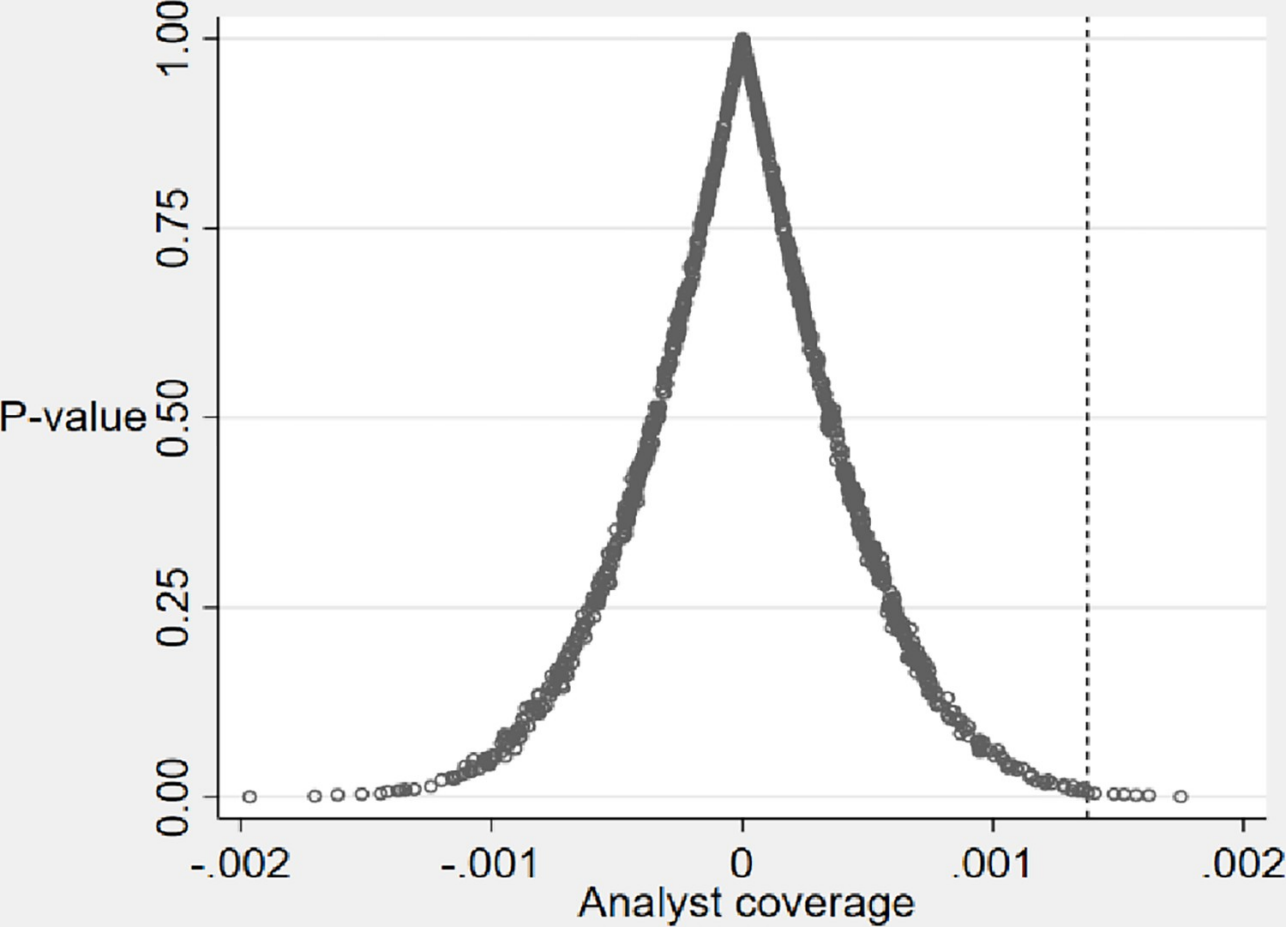
Placebo test.

To exclude interference from unobservable factors, we conducted a placebo test [54]. We extracted all core explanatory variables from the entire sample and randomly assigned them, evidencing the experiment’s randomness and objectivity. Subsequently, we simulated 1000 regression tests; this practice of repeating the experiment multiple times helped to minimize errors and increase the credibility of the results. The test result is shown in Fig 2. The distribution of the estimated coefficients for the random sample is close to a normal distribution with zero mean, corresponding to p-values mostly greater than 0.1. The dashed line in the figure indicates the true estimated coefficient (0.125), which is located in the right-hand tail of the placebo-tested area plot. This suggests that the baseline results are small probability events in a randomized trial and that the facilitating effect of analyst coverage on corporate green transformation does not occur by chance.

#### 4.3.3. Propensity score matching test (PSM)

To alleviate the problem of sample self-selection and missing variables, we used a propensity score matching test. Enterprises that received analyst coverage were treated as the observation group, and enterprises that did not receive analyst coverage were treated as the control group. We used the control variables as matching variables and estimated a logit model. We used one-to-one matching for observations in the range of common support. As shown in column 3 of Table 4, the regression coefficient for *Analyst* was 0.0113 (p<0.01), which is consistent with the previous findings.

### 4.4. Robustness test

#### 4.4.1. Controlling the fixed effect of enterprises

We conducted a re-estimation using a firm fixed-effects model. In column (1) of Table 5, we found that analyst coverage remained significantly and positively associated with enterprise green transition at the 1% level. This further suggests that the conclusions we draw are robust.

**Table 5:** Robustness test.

	(1)	(2)	(3)	(4)	(5)
	Enterprises green transition	Enterprises green transition	Green patent	Enterprises green transition	Enterprises green transition
Analyst coverage	0.0029***		0.2460***	0.0123***	0.0140***
	(0.0008)		(0.0165)	(0.0011)	(0.0015)
Analyst_r		0.0099***			
		(0.0009)			
GDP				-0.0047***	
				(0.0015)	
Op				0.0366***	
				(0.0086)	
Ub				-0.1360***	
				(0.0256)	
DF				0.0003***	
				(0.0001)	
Constant	0.2147***	0.0628***	-0.5361***	0.0963***	0.0413***
	(0.0103)	(0.01124)	(0.1408)	(0.0251)	(0.0130)
Control variables	Yes	Yes	Yes	Yes	Yes
Firm	Yes	No	No	No	No
Industry	No	Yes	Yes	Yes	Yes
Year	Yes	Yes	Yes	Yes	Yes
N	14762	14779	13319	14213	10775
R^2^	0.7357	0.2812	0.3059	0.2924	0.2923
Adjusted R^2^	0.6914	0.2778	0.3023	0.2888	0.2878

Note

*p < 0.1

**p < 0.05

***p < 0.01

#### 4.4.2. Substitution variable

First, we re-estimated the model using an alternative measurement of analyst coverage: the number of research reports *(Analyst_r)* issued by analysts for each firm. In column (2) of Table 5, the regression coefficient for *Analyst_r* was 0.0099 (p<0.01), which is consistent with the above results. Second, we replaced the measurement of enterprise green transition. Yu et al. [55] contended that green innovation is the main motivator for green transitions. Therefore, we used the natural logarithm of green patent applications plus 1 (GP) to measure the level of enterprise green transition. In column (3) of Table 4, the coefficient for analyst coverage was significant and positive, which again supports H1a.

#### 4.4.3. Adding variables

We further controlled for macro-level variables that may affect green transition, including the level of economic development (GDP), the degree of openness to the outside world (Op), and the level of urbanization (Ub), the level of digital financial development (DF). The results showed in column (4) of Table 5 suggested that the conclusions of this study remain robust.

#### 4.4.4. Excluding observations

In the above analysis, our sample includes firm observations with zero analyst coverage. It could be argued that firms with no analyst coverage are intrinsically different from others. However, so far, we have implicitly assumed that the only discrepancy between them is the number of analysts who cover them, not whether they are covered or not. To take this into account, we investigated whether our findings change significantly if we exclude firms with zero analyst coverage. The regression results were presented in columns (5) of Table 5. Our sample is greatly reduced from 14779 to 10775. However, the main results remain qualitatively the same as significant at the 1% level.

## 5. Mechanism tests

Thus far, we have found that analyst coverage can contribute to the green transition of firms by facilitating information transmission and serving as an external regulatory mechanism. There are three possible channels through which it can play these roles: first, enhancing corporate information transparency; second, weakening principal-agent conflicts; and third, increasing corporate environmental legitimacy pressure. We applied the Wen and Ye [56] modified mediating effect test to verify the existence of three mechanisms.

$$\begin{array}{c} EGT_{i,t}=\beta_{0}+\beta_{1}Analyst_{i,t}+\rho_{k}X_{i,t}+\mu_{i}+\gamma_{t}+\varepsilon_{i,t}\\ M_{i,t}=\beta_{0}+\beta_{1}Analyst_{i,t}+\rho_{k}X_{i,t}+\mu_{i}+\gamma_{t}+\varepsilon_{i,t}\\ EGT_{i,t}=\beta_{0}+\beta_{1}Analyst_{i,t}+\beta_{2}M_{i,t}+\rho_{k}X_{i,t}+\mu_{i}+\gamma_{t}+\varepsilon_{i,t} \end{array}$$ (3)

where *M*_*i*,*t*_ represents information transparency, principal-agent conflict, and environmental legitimacy pressure, respectively.

### 5.1. Information transparency, analyst coverage, and enterprise green transitions

Analysts help convey the value of green transition activities, enhance the information transparency of enterprises, and strengthen investors’ “willingness to invest,” thus promoting green transitions. To assess the mediating effect of information transparency, we used the information quality evaluation grade disclosed by the Shenzhen Stock Exchange to measure the information transparency of enterprises [57]. Table 6 shows the results.

**Table 6:** Information transparency, analyst coverage, and enterprise green transition.

	(1)	(2)	(3)
	Enterprises green transition	TC	Enterprises green transition
Analyst coverage	0.0125***	0.0811***	0.0116***
	(0.0011)	(0.0072)	(0.011)
TC			0.01120***
			(0.0017)
Constant	0.0627***	2.6142***	0.0333***
	(0.0112)	(0.0711)	(0.0120)
Control variables	Yes	Yes	Yes
Industry	Yes	Yes	Yes
Year	Yes	Yes	Yes
N	14779	14779	14779
R^2^	0.2818	0.2203	0.2874
Adjusted R^2^	0.2783	0.2166	0.2839
Sobel Z	9.122***		
Mediation Effect	$\frac{ab}{c}=\frac{0.0811\times 0.0112}{0.0125}=7.3\%$		

Note

*p < 0.1

**p < 0.05

***p a.01

As shown in column (2) of Table 6, analyst coverage significantly enhanced information transparency. Column (3) of Table 6 shows the results of a regression of enterprise green transition on analyst coverage and the mediating factor. The regression coefficient for TC was 0.0112 (p<0.01), which indicates that information transparency significantly increased corporate green transitions. Moreover, the regression coefficient for analyst coverage decreased after controlling for TC. The results show that information transparency operated as a mediating channel in this relationship. Meanwhile, the Sobel test confirmed that analyst coverage promoted enterprise green transition by increasing information transparency. The proportion of mediated effects to total effects is 7.3%.

### 5.2. Principal-agent conflict, analyst coverage, and enterprise green transition

To test whether the principal-agent conflict is a channel for analysts to influence the corporate green transition, we used the management expense ratio (MER) to measure the principal-agent conflict of enterprises. The results of our estimation are shown in Table 7.

**Table 7:** Principal-agent conflict, analyst coverage, and enterprise green transition.

	(1)	(2)	(3)
	Enterprises green transition	MER	Enterprises green transition
Analyst coverage	0.0125***	-0.0017***	0.0121***
	(0.0011)	(0.0006)	(0.0011)
MER			-0.2052***
			(0.0223)
Constant	0.0627***	0.1606***	0.0956***
	(0.0112)	(0.0074)	(0.0115)
Control variables	Yes	Yes	Yes
Industry	Yes	Yes	Yes
Year	Yes	Yes	Yes
N	14779	14779	14779
R^2^	0.2818	0.3212	0.2936
Adjusted R^2^	0.2783	0.3180	0.2902
Sobel Z	4.522***		
Mediation Effect	$\frac{ab}{c}=\frac{\left(-0.0017)\times (-0.2052\right)}{0.0125}=2.8\%$		

Note

*p < 0.1

**p < 0.05

***p < 0.01

As shown in column (2) of Table 7, the regression coefficient for analyst coverage was -0.0017 (p<0.01), indicating that it inhibited principal-agent conflict. Combined with the results in column 3, it is clear that principal-agent conflict significantly inhibited enterprise green transition; after controlling for this intermediate variable, the regression coefficient for analyst coverage was smaller. The result demonstrates principal-agent conflict as a mediating channel in this relationship. Further, according to the results of the Sobel test, the statistic was 4.522 and significant at the 1% level, indicating that the mediator effect is effective.

### 5.3. Environmental legitimacy pressure, analyst coverage, and enterprise green transitions

In the current social context of advocating environmental protection, stakeholders and the public are increasingly concerned about environmental governance performance and the green innovation activities of enterprises. Analysts, as information transmission channels in the capital market, can enhance the supervision of listed companies’ business activities by society. This increases corporate awareness of environmental laws and strengthens the environmental legitimacy pressure faced by enterprises, forcing them to make green transitions. Cormier et al. [58] indicated that public media data is an indicator of corporate environmental legitimacy pressure. We draw on related research to measure the environmental legitimacy pressure faced by firms using the convergence of public media coverage. Table 8 shows the results of our estimation.

**Table 8:** Environmental legitimacy pressure, analyst coverage, and enterprise green transition.

	(1)	(2)	(3)
	Enterprises green transition	ELP	Enterprises green transition
Analyst coverage	0.0125***	0.3955***	0.0083***
	(0.0011)	(0.0194)	(0.0011)
ELP			0.0108***
			(0.0010)
Constant	0.0627***	0.8898***	0.0517***
	(0.0112)	(0.1815)	(0.0110)
Control variables	Yes	Yes	Yes
Industry	Yes	Yes	Yes
Year	Yes	Yes	Yes
N	14779	14779	14779
R^2^	0.2818	0.3444	0.3034
Adjusted R^2^	0.2783	0.3311	0.2999
Sobel Z	18.37***		
Mediation Effect	$\frac{ab}{c}=\frac{0.3955\times 0.0108}{0.0125}=34\%$		

Note

*p < 0.1

**p < 0.05

***p < 0.01

As shown in column (2) of Table 8, the regression coefficient for analyst coverage was 0.3955 (p<0.01), indicating that it increased the environmental legitimacy pressure faced by firms. Combined with the regression results in column (3) of Table 8, this indicates that environmental legitimacy pressure increased enterprise green transition. After controlling this intermediate variable, the regression coefficient of analyst coverage was smaller, which passes the Sobel test at least at the significance 1% level, indicating that ELP did mediate the relationship. The proportion of mediated effects to total effects is 34%.

## 6. Heterogeneity test

### 6.1. Enterprise size heterogeneity

Compared with small-scale firms, large-scale firms have greater liquidity for investment transactions. Thus, investors will pay more attention to these types of firms. Moreover, because analysts’ main income source is derived from institutional investors’ split positions and trading commissions, they have stronger incentives to track large-scale firms. This suggests that analyst coverage should have a stronger effect on green transitions among large-scale firms. To compare the effect of analysts on green transition between different firm size groupings, we classified enterprises larger than the median as large-scale enterprises and those smaller than the median as small-scale enterprises. As shown in columns (1) and (2) of Table 9, analyst coverage promoted green transitions among both large-scale and small-scale firms. However, the coefficient was larger for large-scale firms (0.0131, p<0.01) than for small-scale firms (0.0022, p<0.1), and the difference between the two coefficients was statistically significant. These results indicate that analyst coverage had a stronger effect on the promotion of green transitions for large-scale firms.

**Table 9:** Heterogeneity test.

	Large-scale enterprises	Small-scale enterprises	State-owned enterprises	Non-State-owned enterprises
	Enterprises green transition	Enterprises green transition	Enterprises green transition	Enterprises green transition
Analyst coverage	0.0131***	0.0022*	0.0156***	0.0108***
	(0.0016)	(0.0013)	(0.0021)	(0.0013)
_cons	0.0780***	0.1127***	0.0642***	0.0748***
	(0.0155)	(0.0155)	(0.0234)	(0.0128)
Experience P-value	^0.000^ ***		^0.000^ ***	
Control variables	Yes	Yes	Yes	Yes
Industry	Yes	Yes	Yes	Yes
Year	Yes	Yes	Yes	Yes
N	8065	6710	4345	10433
R^2^	0.2470	0.1873	0.3109	0.2461
Adjusted R^2^	0.2409	0.1795	0.3019	0.2412

Note

*p < 0.1

**p < 0.05

*ap < 0.01

### 6.2. Ownership form heterogeneity

There are unique variations in ownership forms in the Chinese capital market. Different forms of ownership can lead to great differences in the management efficiency and governance structures of enterprises. We divided the sample into state-owned enterprises and non-state-owned enterprises and re-estimated the model. As shown in columns (3) and (4) of Table 9, the coefficients for analyst coverage were significant at the 1% level for both state-owned and non-state-owned enterprises. However, the effect was smaller for non-state-owned enterprises, and the difference between the two coefficients was statistically significant. This indicates that the promotional effect of analyst coverage on corporate green transition was stronger among state-owned enterprises. This could be due to the dependence of state-owned enterprises on government support for their business activities and their natural advantages in accessing resources, which leads to inefficiencies and greater principal-agent problems. Thus, analysts can play a greater supervisory role for state-owned enterprises, promoting their green transitions.

## 7. Conclusions and recommendations

China’s “double carbon” policy seeks to balance economic growth with pollution and carbon reduction to maximize economic and environmental benefits. The multi-dimensional transition of the economic system is the only way to coordinate economic growth and environmental protection. As the micro-foundation of the economic system, enterprise green transition is related to the overall green and low-carbon transition of the economy and society. Moreover, China is actively promoting capital market reform to encourage green and high-quality economic development. Given that analysts play an important role in capital markets, it is necessary to explore how analysts shape them and the influencing mechanism behind this impact.

Accordingly, we used entropy weighting to develop an index of corporate green transitions with four dimensions: green innovation, green production, green governance performance, and social responsibility. Moreover, we examined the influence of analyst coverage on green transitions by manufacturing enterprises. Our study found that analyst coverage dramatically increased enterprise green transition; the more analysts following an enterprise, the more likely the enterprise is to undergo green transition. We attribute this result to the information transmission effect and external supervision effect of analysts, which override the pressure effect. The enhancement of the information transmission effect improves information transparency and enhances investors’ value cognition. Meanwhile, the external supervision effect reduces the cost of a management agency and increases the pressure on environmental legitimacy. Our study highlights analysts’ significant role in firms’ green transitions and strengthens practitioners’ understandings of external monitoring beyond internal information governance. Subsequently, considering factors such as the scale of enterprises and the nature of property rights, we conducted a cross-sectional regression analysis of the main effects. The results indicate that in large-scale and state-owned enterprises, analyst coverage plays an obvious role in promoting green transitions.

Compared to the results of previous studies, our findings support the idea that the information and monitoring effects reported by analysts dominate corporate investment strategies [17, 35, 59]. Meanwhile, it is also important to note that environmental disclosure [60, 61], agency cost [62, 63], and environmental legitimacy pressures [64, 65] are important factors influencing firms’ green transitions. However, while our study reveals that analyst coverage has a positive effect on enterprise green transition, we cannot rule out its possible negative effect on green transition, as suggested by the market pressure hypothesis [31, 66]—our evidence reflects only the net effect of analyst coverage on green transition.

Based on the above conclusions, we recommend that policymakers seeking to facilitate corporate green transitions formulate policies along the following lines. First, the government should promote “standardization, transparency, openness, vitality, and resilience” to optimize the capital market and highlight the role of intermediaries. Second, the government should encourage analysts to expand their coverage of firms’ green transitions to enlarge the promotional effect of analyst coverage on green transitions. Third, policymakers should emphasize the creation of effective policies to promote positive externalities for analysts. For example, policymakers should ensure that information disclosure channels for environmental governance are accessible and effective.

Our study had some limitations, which point to several directions for further research. First, we only provide insights on how the number of analysts promotes the green transitions of enterprises. Future research could also consider heterogeneities in analyst coverage characteristics, such as the depth of analyst coverage and optimism, and how these differences may affect the green transitions of enterprises. Second, our results are highly dependent on China’s institutional and market characteristics, which are characterized by its status as an emerging market country—similar studies conducted outside this context may yield different results; for example, He et al. [31] validate the “dark side” of analyst coverage on corporate innovation based on U.S. market data. Therefore, we believe that future research must explore the different impacts that analyst coverage may have on firms’ strategies in light of a wider range of contextual factors. Third, our sample is limited to Chinese manufacturing firms, which are facing severe environmental problems, scrutiny, and legitimacy pressures; therefore, future studies should include a broader range of organizations, such as family-owned firms or public organizations, to ensure the robustness and validity of the findings.

## Supporting information

S1 Data (RAR)

## References

[1] Guo K, Tian X. Accelerate the green transformation of Chinese industrial development mode: achievements, challenges and paths. Economic Review Journal. 2023; 1: 8–16. 10.16528/j.cnki.22-1054/f.202301008

[2] Li P. A study on the green transformation of Chinese industry. China Industrial Economics. 2011; 4: 5–14. 10.19581/j.cnki.ciejournal.2011.04.001

[3] Morgera E, Savaresi A. A conceptual and legal perspective on the green economy. Review of European, Comparative and International Environmental Law. 2013; 22: 14–28. 10.1111/reel.12016

[4] Feng Y, Ning M, Lei Y, et al. Defending blue sky in China: effectiveness of the “air pollution prevention and control action plan” on air quality improvements from 2013 to 2017. Journal of Environmental Management. 2019; 252: 109603. doi: 10.1016/j.jenvman.2019.109603 31586746

[5] Shao Y, Chen Z. Can government subsidies promote the green technology innovation transformation? Evidence from Chinese listed companies. Economic Analysis and Policy. 2022; 74: 716–727. 10.1016/j.eap.2022.03.020

[6] Wang Z, Li X, Xue X, Liu Y. More government subsidies, more green innovation? The evidence from Chinese new energy vehicle enterprises. Renewable Energy. 2022; 197: 11−21. 10.1016/j.renene.2022.07.086

[7] Liu B, Cifuentes-Faura J, Ding J, et al. Toward carbon neutrality: How will environmental regulatory policies affect corporate green innovation? Economic Analysis and Policy. 2023; 80: 1006–1020. 10.1016/j.eap.2023.09.019

[8] Zhang D. Environmental regulation, green innovation, and export product quality: What is the role of greenwashing? International Review of Financial Analysis. 2022; 83: 102311. 10.1016/j.irfa.2022.102311

[9] Li X., Hamblin D. Factors impacting on cleaner production: case studies of Chinese pharmaceutical manufacturers in Tianjin, China. Journal of Cleaner Production. 2016; 131: 121–132. 10.1016/j.jclepro.2016.05.066

[10] Liu F, Sim J Y, Sun H, Edziah B K, et, al. Assessing the role of economic globalization on energy efficiency: Evidence from a global perspective. China Economic Review. 2023; 77: 101897. 10.1016/j.chieco.2022.101897.

[11] Huang W, Yuan T. Substantive transformation and upgrading or strategic policy arbitrage—Research on the Impact of Green Industry Policy on Green M&As of Industrial Enterprises. Journal of Shanxi University of Finance and Economics. 2021; 43(3): 56–67. 10.13781/j.cnki.1007-9556.2021.03.005

[12] Wu H, Li Y, Hao Y, Ren S, et al. Environmental decentralization, local government competition, and regional green development: Evidence from China. Science of the Total Environment. 2020; 708: 135085. doi: 10.1016/j.scitotenv.2019.135085 31812397

[13] Gallen T S, Winston C. Transportation capital and its effects on the U.S. economy: A general equilibrium approach. Journal of Macroeconomics. 2021; 69: 103334. 10.1016/j.jmacro.2021.103334

[14] Petroni G., Bigliardi B, Galati F. Rethinking the porter hypothesis: The underappreciated importance of value appropriation and pollution intensity. Review of Policy Research. 2019; 36: 121–140. 10.1111/ropr.12317

[15] Qian C, Lu Y, Yu Y. Financial analyst coverage and corporate social performance: Evidence from natural experiments. Strategic Management Journal. 2019; 40(1): 2271–2286. 10.1002/smj.3066

[16] Gentry R J, Shen W. The impacts of performance relative to analyst forecasts and analyst coverage on firm R&D intensity. Strategic Management Journal. 2013; 34(1): 121–130. 10.1002/smj.1997

[17] Guo B, Pérez-Castrillo D, Toldrà-Simats A. Firms’ innovation strategy under the shadow of analyst coverage. Journal of Financial Economics. 2019; 131(2): 456–483. 10.1016/j.jfineco.2018.08.005

[18] Lin M, Wu C, Chiang M. Investor attention and information diffusion from analyst coverage. International Review of Financial Analysis. 2014; 20: 235–246. 10.1016/j.irfa.2014.03.006

[19] Mehran H, Peristiani S. Financial visibility and the decision to go private. Review of Financial Studies. 2010; 23(2): 519–547. 10.1093/rfs/hhp044

[20] Mola S, Rau P R, Khorana A. Is there life after the complete loss of analyst coverage? The Accounting Review. 2013; 88(2): 667–705. 10.2308/accr-50330

[21] Kim J B, Lu L Y, Yu Y. Analyst coverage and expected crash risk: Evidence from exogenous changes in analyst coverage. The Accounting Review. 2019; 94(4): 345–364. 10.2308/accr-52280

[22] Dyck A, Lins K V, Roth L, et al. Do institutional investors drive corporate social responsibility? International evidence. Journal of Financial Economics. 2019; 131(3): 693–714. 10.1016/j.jfineco.2018.08.013

[23] Chen T, Harford J, Lin C. Do analysts matter for governance? Evidence from natural experiments. Journal of Financial Economics. 2015; 115(2): 383–410. 10.1016/j.jfineco.2014.10.002

[24] Yu F. Analyst coverage and earnings management. Journal of Financial Economics. 2008; 88(2): 245–271. 10.1016/j.jfineco.2007.05.008

[25] Israelsen R D. Does common analyst coverage explain excess comovement? Journal of Financial and Quantitative Analysis. 2016; 51(4): 1193–1229. 10.1017/S002210901600051X

[26] Callao S, Jarne J I. Analysts’ forecasts as an incentive for earnings management. Spanish Journal of Finance and Accounting‒Revista Espanola de Financiacion Y Contabilidad. 2018; 47(1): 124–155. 10.1080/02102412.2017.1371977

[27] Asquith P, Mikhail M B, Au A S. Information content of equity analyst reports. Journal of Financial Economics. 2005; 75(2): 245–282. 10.1016/j.jfineco.2004.01.002

[28] Hsieh S J, Su Y. The effect of financial analysts on the economic implications of disclosed lease information-a note. Journal of Applied Accounting Research. 2022; 23(2): 340–361. 10.1108/JAAR-08-2020-0175.

[29] Lin Z A, Qc B, Sy C, et al. The role of analysts in negative information production and disclosure: Evidence from short selling deregulation in an emerging market. International Review Economics & Finance. 2021; 73: 391–406. 10.1016/j. iref.2021.01.016.

[30] Amairi H, Gallali M, Sassi S. Market pressure and cost of equity: Revisited. Finance Research Letters. 2022; 47: 102749. 10.1016/j.frl.2022.102749

[31] He J, Tian X. The dark side of analyst coverage: The case of innovation. Journal of Financial Economics. 2013; 109(3): 856–878. 10.1016/j.jfineco.2013.04.001

[32] Liang H, Wang Z, Niu R. Do environmental regulations promote the green transformation of high polluters? Applied Economics Letters. 2023; 30 (7): 927–931. https://doi. org/10.1080/13504851.2022.2030034.

[33] Wang J, Liu Y, Wang W, et al. How does digital transformation drive green total factor productivity? Evidence from Chinese listed enterprises, Journal of Cleaner Production. 2023; 406: 136954. 10.1016/j.jclepro.2023.136954

[34] Chang X, Dasgupta S, Hilary G. Analyst coverage and financing decisions. The Journal of Finance. 2006; 61(6): 3009–3048. 10.1111/j.1540-6261.2006.01010.x

[35] To T Y, Navone M, Wu E. Analyst coverage and the quality of corporate investment decisions. Journal of Corporate Finance. 2018; 51: 164–181. 10.1016/j.jcorpfin.2018.06.001

[36] Allen A, Francis B B, Wu Q, Zhao Y. Analyst coverage and corporate tax aggressiveness. Journal of Banking & Finance. 2016; 73: 84–98. 10.1016/j.jbankfin.2016.09.004

[37] Li S. Legitimacy pursuit, profit-seeking tendency and the strategic operations of local governments’ environmental governance. China Population, Resources and Environment. 2020; 30(12): 137–146. 10.12062/cpre.20200944

[38] Moyer R, Chatfield R, Sisneros P. Analyst monitoring activity: Agency costs and information demands. Journal of Financial Quantitative Analysis. 1989; 24(4): 503–512. 10.2307/2330982

[39] Hou J, Zhao S, Yang H. Individual analysts, stock return synchronicity and information efficiency. International Review of Financial Analysis. 2020; 71: 101513. 10.1016/j.irfa.2020.101513

[40] Zhou L, Zhang X. R&D investment and stock price crash risk: An empirical evidence from Chinese A-share listed companies. On Economic Problems. 2020; 7: 67–75. 10.16011/j.cnki.jjwt.2020.07.010

[41] Healy M, Palepu K. Information asymmetry, corporate disclosure, and the capital markets: A review of the empirical disclosure literature. Journal of Accounting & Economics. 2001; 31: 405–440. 10.2139/ssrn.258514

[42] Wei Z, Gu M. Corporate legitimacy and green performance in transformation economy. Management Review. 2015; 27(4): 76–84. 10.14120/j.cnki.cn11-5057/f.2015.04.009

[43] Huang M, Li M, Liao Z. Do politically connected CEOs promote Chinese listed industrial firms’ green innovation? The mediating role of external governance environments. Journal of Cleaner Production. 2021; 278: 123634. 10.1016/j.jclepro.2020.123634

[44] Hui K W, Liu A Z, Zhang Y. The rewards for meeting or beating managers’ own earnings guidance. Accounting Horizons. 2021; 32(1): 87–103. 10.2308/HORIZONS-16-059

[45] Mcgowan P. Use of effectuation by established micro businesses: short-term gain, long-term pain? Journal of Business & Industrial Marketing. 2021; 36(1): 60–71. 10.1108/JBIM-01-2020-0055

[46] Martens T, Sextroh C. Analyst coverage overlaps and interfirm information spillovers. Journal of Accounting Research. 2021; 59(4): 1425–1480. 10.1111/1475-679X.12391

[47] Lin B, Zhou Y. Measuring the green economic growth in China: Influencing factors and policy perspectives. Energy. 2022; 241: 122518. 10.1016/j.energy.2021.122518

[48] Lu Q, Wu Q, Zhou Y, et al. Analysis on the assessment of greenery transformation-upgrading of industry in Guangdong. China Population, Resources and Environment. 2013; 23: 34–41. 10.3969/j.issn.1002-2104.2013.07.006

[49] Sun C, Zhang W. Outward foreign direct investment and enterprise green transformation: an empirical study based on the micro data of Chinese enterprises. China Population, Resources and Environment. 2022; 32: 79–91. 10.12062/cpre.20220432

[50] Santos B M D, Godoy L P, Campos L M S. Performance evaluation of green suppliers using entropy-TOPSIS-F. Journal of Cleaner Production. 2019; 207: 498–509. 10.1016/j.jclepro.2018.09.235

[51] Lewis B, Walls J, Dowell G. Difference in degrees: CEO characteristics and firm environmental disclosure. Strategy Management Journal. 2014; 35(5): 712–722. 10.1002/smj.2127

[52] Liu X, Zhang S. Chinese enterprise green transformation: Target pattern, obstacles and countermeasures. China Population, Resources and Environment. 2015; 25(6): 1–4. 10.3969/j.issn.1002-2104.2015.06.001

[53] Ding X, Xu Z, Petrovskaya M V, et al. Exploring the impact mechanism of executives’ environmental attention on corporate green transformation: Evidence from the textual analysis of Chinese companies’ management discussion and analysis. Environmental Science and Pollution Research. 2023; 30(31): 76640–76659. doi: 10.1007/s11356-023-27725-4 37237116 PMC10219686

[54] Zhang C, Chen D. Do environmental, social, and governance scores improve green innovation? Empirical evidence from Chinese-listed companies. PLoS ONE. 2023; 18(5): e0279220. doi: 10.1371/journal.pone.0279220 37228130 PMC10212161

[55] Yu L, Zhang, Bi, Q. Can the reform of environmental protection fee-to-tax promote the green transformation of high-polluting enterprises?——evidence from quasi-natural experiments implemented in accordance with the environmental protection tax law. China Population, Resources and Environment. 2021; 31: 109–118. 10.12062/cpre.20200703

[56] Wen Z, Ye B. Analyses of Mediating Effects: The Development of Methods and Models. Advanced in Psychological Science. 2014; 22(5): 731–745. 10.3724/SP.J.1042.2014.00731

[57] Xie G, Hao Y, Li S. Industry concentration, analysts’ industry expertise and forecast accuracy. Foreign Economics & Management. 2019; 41: 125–138. 10.16538/j.cnki.fem.2019.02.010

[58] Cormier D, Magnan M. The economic relevance of environmental disclosure and its impact on corporate legitimacy: An empirical investigation. Business Strategy and the Environment. 2015; 24(6): 431–445. 10.1002/bse.1829

[59] Zhang P, Wang Y. The bright side of analyst coverage on corporate innovation: Evidence from China. International Review of Financial Analysis. 2023; 89: 102791. 10.1016/j.irfa.2023.102791

[60] Feng Y, Wang X, Liang Z. How does environmental information disclosure affect economic development and haze pollution in Chinese cities? The mediating role of green technology innovation. Science of the Total Environment. 2021; 775: 145811. doi: 10.1016/j.scitotenv.2021.145811 33631595

[61] Xiang X, Liu C, Yang M, et al. Confession or justification: The effects of environmental disclosure on corporate green innovation in China. Corporate Social Responsibility and Environmental Management. 2020; 27(6): 2735–2750. 10.1002/csr.1998

[62] Yermack D. Higher market valuation of companies with a small board of directors. Journal of Financial Economics. 1996; 40(3): 185–211. 10.1016/0304-405x(95)00844-5

[63] Rashid A. CEO duality and agency cost: evidence from Bangladesh. Journal of Management & Governance. 2013; 17(4): 989–1008. 10.1007/s10997-012-9213-x

[64] Li D, Zheng M, Cao C, et al. The impact of legitimacy pressure and corporate profitability on green innovation: Evidence from China top 100. Journal of Cleaner Production. 2017; 141: 41–49. 10.1016/j.jclepro.2016.08.123

[65] Zhou M, Govindan K, Xie X, et al. How to drive green innovation in China’s mining enterprises? Under the perspective of environmental legitimacy and green absorptive capacity. Resources Policy. 2021; 72: 102038. 10.1016/j.resourpol.2021.102038

[66] Amiram D, Bozanic Z, Cox J D, et al. Financial reporting fraud and other forms of misconduct: A multidisciplinary review of the literature. Review of Accounting Studies. 2018; 23(2): 732–783. 10.1007/s11142-017-9435-x

